# MB3W1 is an orthotopic xenograft model for anaplastic medulloblastoma displaying cancer stem cell- and Group 3-properties

**DOI:** 10.1186/s12885-016-2170-z

**Published:** 2016-02-17

**Authors:** Sebastian Dietl, Stefanie Schwinn, Susanne Dietl, Simone Riedel, Frank Deinlein, Stefan Rutkowski, Andre O. von Bueren, Jürgen Krauss, Tilmann Schweitzer, Giles H. Vince, Daniel Picard, Matthias Eyrich, Andreas Rosenwald, Vijay Ramaswamy, Michael D. Taylor, Marc Remke, Camelia M. Monoranu, Andreas Beilhack, Paul G. Schlegel, Matthias Wölfl

**Affiliations:** University Children’s Hospital, Pediatric Oncology, Hematology and Stem Cell Transplantation, University of Würzburg, Würzburg, Germany; Department of Surgery II, University of Würzburg, Würzburg, Germany; Interdisciplinary Center for Clinical Research Laboratory (IZKF Würzburg), Department of Internal Medicine II, University of Würzburg, Würzburg, Germany; Department of Pediatric Oncology, University Medical Center Hamburg-Eppendorf, Hamburg, Germany; Department of Pediatrics and Adolescent Medicine, Division of Pediatric Hematology and Oncology, University Hospital of Geneva, Geneva, Switzerland; Department of Neurosurgery, University of Würzburg, Würzburg, Germany; Department of Pediatric Oncology, Hematology and Clinical Immunology / Department of Neuropathology, Heinrich Heine University, Düsseldorf, Germany; Institute of Pathology, University of Würzburg, Würzburg, Germany; Division of Neurosurgery, Arthur and Sonia Labatt Brain Tumor Research Centre, Hospital for Sick Children, Toronto, Canada; Comprehensive Cancer Center Mainfranken, University of Würzburg, Würzburg, Germany

**Keywords:** Anaplastic medulloblastoma, Group 3, Orthotopic xenograft, Cancer stem cells, Animal model, Brain tumor, Children

## Abstract

**Background:**

Medulloblastoma is the most common malignant brain tumor in children and can be divided in different molecular subgroups. Patients whose tumor is classified as a Group 3 tumor have a dismal prognosis. However only very few tumor models are available for this subgroup.

**Methods:**

We established a robust orthotopic xenograft model with a cell line derived from the malignant pleural effusions of a child suffering from a Group 3 medulloblastoma.

**Results:**

Besides classical characteristics of this tumor subgroup, the cells display cancer stem cell characteristics including neurosphere formation, multilineage differentiation, CD133/CD15 expression, high ALDH-activity and high tumorigenicity in immunocompromised mice with xenografts exactly recapitulating the original tumor architecture.

**Conclusions:**

This model using unmanipulated, human medulloblastoma cells will enable translational research, specifically focused on Group 3 medulloblastoma.

**Electronic supplementary material:**

The online version of this article (doi:10.1186/s12885-016-2170-z) contains supplementary material, which is available to authorized users.

## Background

Medulloblastoma is the most common malignant brain tumor in childhood [1]. The current standard of care consists of multimodal age- and stage-adapted therapy including surgical resection, irradiation and chemotherapy. The approach significantly increased survival rates over the last decades, but a subset of tumors with a still devastating prognosis remains. These aggressive tumors do not respond even to high intensity treatment regimens [2]. Indicators of poor prognosis are large cell/anaplastic (LCA) histology [3–6], metastasis [7, 8], *MYC* amplification [3–5, 8–10], *TP53* alteration [11, 12] and gain of chromosome 17q [9].

Gene expression analysis clearly defines molecular subgroups with distinct biological characteristics. These subgroups differ in their cellular origins, activation pathways and clinical/pathological characteristics [13–17]. Therefore medulloblastoma cannot be considered as one single disease entity. There is a consensus that four different main molecular subgroups of medulloblastoma exist: WNT, SHH, Group 3 and Group 4 [18]. For WNT and SHH the driving pathways are known and well-validated mouse models are established [18–22]. For Group 3 and 4 tumors data are more limited, also due to the lack of appropriate animal models. As Group 3 tumors have the worst prognosis among the identified subgroups, there is a clear need for reliable tumor models. This subgroup of medulloblastoma almost only occurs in infants and children, particularly in males [23, 24]. Furthermore, it is marked by an extremely high dissemination tendency into the cerebrospinal fluid (CSF). Genetic alterations are found frequently, such as gain of chromosome 17q and amplification of the *MYC* oncogene. In fact, in most cases amplification of the *MYC* oncogene seems to be restricted to this group and associated with poor clinical outcome [18, 23, 24]. Two recent studies focus on syngenic mouse models engineering Myc-overexpressing cerebellar cells [25, 26]. Pei et al. introduced *Myc* into CD133^+^ cells of the cerebellar white matter and Kawauchi et al. into granule neuron precursors. In combination with p53 blockade both models led to the formation of highly aggressive medulloblastomas recapitulating human *MYC*-driven Group 3 medulloblastoma. Furthermore Stearns et al. evaluated xenograft models of the medulloblastoma cell lines DAOY and UW228, demonstrating that Myc overexpression was required to achieve tumor engraftment of UW228 cells which was linked to anaplastic histology [27]. Two other groups also established murine medulloblastoma models with anaplastic characteristics, but in these tumors overexpression of MYCN is a key characteristic [28, 29].

The cancer stem cell hypothesis suggests that within one tumor a hierarchy of tumor cells exist: most cancerous cells will not have the propensity to create new tumors by themselves, whereas the few tumor-initiating cells are the founding cells of an arising tumor. These undifferentiated self-renewing cells are the propagating pool responsible for tumor growth [30]. Cancer stem cells (CSC) seem to be a major cause for tumor aggressiveness and relapse because of their high radio- and chemoresistance [31, 32]. Therefore studying this cell population could be a reasonable and promising approach for the understanding of tumor pathogenesis and for the development of new therapies [33, 34].

For medulloblastoma several lines of evidence support the CSC hypothesis [33–36]. Although questions about the frequency of such cells, their origin and the exact phenotypical and functional characteristics remain. Experimentally the capacity to exactly recapitulate the original tumor architecture in xenograft models, with tumors arising from very few cells, is a strong indicator of CSC properties [37]. Clinically the role of these aggressive cells, e.g. with regard to metastasis, is even less clear.

Here we describe a case of a Group 3 medulloblastoma with an unusual clinical occurrence of extracranial metastasis of tumor cells displaying predominantly CSC characteristics. When transplanted as an orthotopic murine xenograft this anaplastic medulloblastoma demonstrates many characteristics reported for CSC as well as for the highly aggressive Group 3 medulloblastoma.

## Methods

### Clinical case

Diagnostics and treatment of the patient were conducted at the University Hospital of Würzburg according to HIT 2000 and HIT-Rez 2005 trial of the German Society of Pediatric Oncology and Hematology. These therapeutic multi-center studies had been approved by the local ethical committee of the University Hospital Würzburg and Bonn (No. 73/00 (Würzburg) for HIT 2000 and No. 105/05 (Bonn) for HIT-Rez 2005) and include terms regarding the use of tumor material for additional studies. The guardians provided written consent for participation of their child on the clinical study. The patient’s parents consented in writing to the analysis of the tumor cells based on an individual decision due to the exceptional clinical course, which is in file along with the medical case documentation. This written consent includes extensive characterization, culture and storage of the tumor cells and establishment of a stable tumor cell line. It also includes genetic characterization and genetic alteration (such as lentiviral transduction) and use of the tumor cells in animal models.

### Tumor cell isolation and cell culture

Tumor cells from the malignant pleural effusions were isolated by performing a Ficoll gradient. Cells were directly propagated using DMEM (GIBCO) supplemented with 10 % foetal bovine serum (PAA), 40 U/ml penicillin (PAA) and 40 μg/ml streptomycin (PAA) for 4 days. After that time point cells were transferred into serum-free DMEM/F12 (GIBCO) containing 20 ng/ml basic fibroblast growth factor (bFGF), (PEPROTECH), 20 ng/ml epidermal growth factor (EGF), (PEPROTECH), 2 % B-27 supplement (GIBCO), 1 % MEM Vitamins (GIBCO), 40 U/ml penicillin (PAA) and 40 μg/ml streptomycin (PAA) and long-term cultured under that conditions. For differentiation, cells were again cultured in serum-containing medium.

### Tumor cell lines

For comparative assays, we used the following tumor cell lines: The glioblastoma cell lines R11 and R28 have been described to have CSC characteristics and were kindly provided by Drs. Beier D and Beier CP (University of Regensburg, now Odense, Denmark). The melanoma cell line FM88 was kindly provided by Dr. Becker C (now University of Essen). MCF7 is a breast carcinoma cell line, kindly provided by Dr. Wischhusen J (University Hospital of Würzburg). U251 and U373 are glioma cell lines, kindly provided by Dr. Hagemann (University Hospital of Würzburg).

### Proliferation assay

Single tumor cells from in serum-free medium cultured neurospheres or from the adherent phase of in serum-containing medium cultured cells were obtained by mechanical dissociation or enzymatic detachment. Triplicates of viable cells were plated in 24 well microplates at densities of 2 × 10^5^ cells/well and propagated in 1 ml/well. After 3 days fresh medium was added. Either serum-containing medium or serum-free medium was used. Every day a triplicate of wells was counted to examine cell proliferation.

### Flow cytometry

Cells were mechanically dissociated to obtain single cell suspensions. After centrifugation cells were resuspended in CliniMACS PBS/EDTA buffer (Miltenyi Biotec) with 0,5 % human serum (PAA). Before staining with fluorochrome conjugated antibodies, Fc receptors were blocked with FcR Blocking Reagent (Miltenyi Biotec). Antibody staining was conducted with CD133/1 and CD133/2 (Miltenyi Biotec, clones AC133 and 293C3) and anti-CD15-antibody (BD, clone MMA) according to the manufacturer’s protocols. Acquisition was performed on a FACS Canto II (BD Biosciences). Dead cells were excluded by 7-AAD (BD Biosciences) staining. Expression of aldehyde dehydrogenase (ALDH) was examined using the ALDEFLUOR kit (STEMCELL Technologies) according to the manufacturer’s protocol.

### Magnetic activated cell sorting

Cells were sorted for CD133/1 expression using the CD133 MicroBead Kit (Miltenyi Biotec). First cells were mechanically dissociated and centrifuged. After resuspension in 300 μl CliniMACS PBS/EDTA buffer with 0.5 % human serum, 100 μl FcR Blocking Reagent and next 100 μl CD133/1 MicroBeads were added. Cells were incubated for 30 min at 4 °C and another 5 min after addition of 50 μl of CD133/2 (Miltenyi Biotec). Next cells were washed and separated using MACS LS columns (Miltenyi Biotec). To achieve higher purities two additional consecutive column runs were performed.

### Lentiviral transduction

Cells were lentivirally transduced with a vector encoding firefly luciferase (FLuc) and enhanced green fluorescent protein (eGFP) as described previously [38]. Transduced cells were enriched by sorting for eGFP expression.

### Animals and orthotopic xenotransplantation

Permission for animal experiments were obtained from the institutional animal care committee for the University Hospital Würzburg. All animal experiments were performed in accordance with national guidelines and regulations and with approval of the district government. Female NOD.CB17-*Prkdc*^*scid*^/J (NOD/SCID) mice were purchased from The Jackson Laboratory and housed under specific pathogen free conditions. Single cell suspensions were prepared either by mechanical disruption or enzymatical detachment, where necessary. Cell numbers were adjusted in culture medium by serial dilution, calculated for an inoculation volume of 3 μl. Cells were orthotopically injected into the brains of 10–13 week-old anesthetized NOD/SCID mice using a stereotaxic instrument (David Kopf Instruments) and a Hamilton syringe with a 26 G needle (Hamilton Company), injecting at defined coordinates: two injection sites were evaluated: for supratentorial inoculation cells were injected in the dorsolateral thalamus, for infratentorial inoculation cells were injected in the right cerebellum. Subsequently, mice were checked daily using bioluminescence imaging (BLI). Survival was defined as the time from transplantation until an early humane endpoint when mice were sacrificed because they showed first symptoms of disease.

### In vivo BLI

Mice were injected intraperitoneally with a mixture of esketamine (80 mg/kg, Pfizer), xylazine (16 mg/kg, CP-Pharma) and D-luciferin (300 mg/kg, Biosynth). 10 min after injection animals were imaged using an IVIS Spectrum imaging system as previously described (Caliper Life Sciences) [39]. Imaging data were analyzed with Living Image 4.0 (Caliper-Xenogen) and Prism 5 software (GraphPad).

### Cytogenetic analysis

Cell cycle arrest was induced by Colcemid (GIBCO). Cells were treated with 0.075 M KCl and fixed in 3:1 alcohol:acetic acid. For karyotyping cells were dried on glass slides and then incubated in 500 μg/ml trypsin (SERVA) for 20 s and subsequently stained in 5 % Giemsa solution for 6 min. For FISH analysis the Vysis LSI MYC/CEP 8 probes, the PathVysion HER-2 DNA and the Vysis MYC Break Apart Rearrangement FISH Probe Kits (Abbott Molecular) according to the manufacturer’s protocols were used: After FISH probes were added, specimens were heat denaturated and incubated at 37 °C over night for hybridization of FISH probes with DNA. Specimens were then washed and mounted with VECTASHIELD Mounting Medium with DAPI (Vector). For visualization the Ikaros and Isis systems (MetaSystems) were used.

### Nanostring analysis

Nanostring analysis was performed on RNA extracted from an early and late passage of the MB3W1 cells according to the methods recently described [40]. Heatmaps were created using the GenePattern software.

### Histopathology and immunohistochemistry

Brains of sacrificed mice were immediately formaldehyde (MERCK) fixed and paraffin (Leica Biosystems) embedded. Specimens were sectioned at a thickness of 3 μm. Histopathology was evaluated by staining sections with a standard Hematoxylin Eosin (HE) protocol. Cytospin preparations were performed at 55 g for 5 min and stained with Pappenheim or HE solutions.

For immunohistochemistry antigen retrieval was conducted with heat induced epitope retrieval using citrate buffer at pH 6.0 (almost all stainings) or with Tris/EDTA buffer at pH 9.0 (CD133 staining). Incubation with the following primary antibodies was performed over night at 4 °C: ßIII-Tubulin (Abcam, ab18207; 1:500), CD99 (DAKO, clone 12E7; 1:200), CD133/1 (Miltenyi, clone AC133; 1:40), GFAP (Millipore, AB5804; 1:6000), INI-1 (BD, clone 25/BAF47; 1:100), Ki-67 (dianova, M501; 1:80), Nestin (Millipore, clone 10C2 1:200), Olig2 (LINARIS, BHU0409, 1:100), p53 (DAKO, clone DO-7; 1:100), Synaptophysin (DAKO, clone SY38; 1:80) and Vimentin (DAKO, clone V9; 1:4000). Immunodetection was performed with the MultiLink HRP kit (BioGenex) and DAB (Dako). Specific antigen recognition was tested by using positive and negative controls.

For immunohistochemical analysis of cytospins the APAAP method was used as a standard method. Briefly, cells were spun onto glas slides and fixed with methanol-acetone. After washing in TRIS-buffer, CD133/1 (Miltenyi, clone AC133) was added onto the slides. Staining control consisted of samples stained with the same procedure, but omitting the primary antibody. After 30 min of incubation at room temperature (RT), slides were washed and incubated with the secondary reagent (rabbit-anti-mouse-antibody, DAKO) for 30 min at RT. After additional washing the APAAP-immunocomplex (tertiary reagent, DAKO) was added for 30 min at, followed by additional wash-steps and incubation with APAAP-reaction solution for 30 min at RT (on a shaker). After additional washing steps, slides were incubated with Haemalaun solution for 2 min, before the slides were finally washed and covered with glass cover slips.

## Results

### Clinical case

**Fig. 1:**
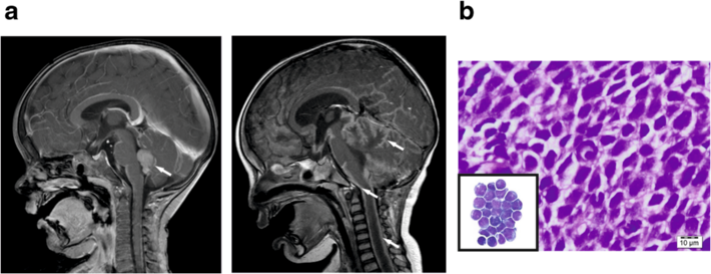
Illustration of the clinical case. **a**. Sagittal cranial MRI of the 22-month-old boy showing the initial tumor in the 4th ventricle (left, *single arrow*) and signs of massive meningeosis 3 weeks later (right, *multiple arrows*). **b**. HE staining of the initial tumor showing typical anaplastic morphology of the tumor cells. *Insert*: medulloblastoma cells from the CSF (Pappenheim-staining)

A 22-month-old boy presented with rapidly progressive gait disorder. Magnetic resonance imaging (MRI) revealed a cerebellar tumor arising from the bottom of the 4th ventricle. Cytology from the CSF was positive for malignant cells. Immediate tumor resection was performed and the diagnosis was confirmed as anaplastic medulloblastoma (Fig. 1a and b). Atypical teratoid/rhabdoid tumor (AT/RT) and Ewing sarcoma (EWS) was excluded based on maintained INI1 expression and absence of the EWS/FLI1 translocation and CD99 expression. Reference pathology (Prof. T. Pietsch, Bonn) confirmed the histopathological diagnosis and determined *MYC* amplification in the original tumor sample. Postoperative MRI showed no residual tumor, but signs of meningeosis. In the days following surgery the child developed intracranial hypertension requiring liquor drainage and a ventriculoperitoneal shunt. Three weeks after resection the boy started to developed signs of brain stem incarceration with brain stem areflexia. MRI revealed a massive increase of the leptomeningeal spread with compression of the brain stem (Fig. 1a, right picture). Emergency cranial irradiation was initiated (initially 3 Gy/day, followed by 2 Gy/day) and subsequently extended to the entire neural axis (total dose: tumor region 53 Gy, cranium 29 Gy, spine 32 Gy). Irradiation-induced partial regression of the leptomeningeal spread was maintained by chemotherapy including lomustine, vincristine and cisplatin (later cyclophosphamide) according to the german treatment optimization study HIT 2000. Nine months after diagnosis the tumor relapsed in the former tumor bed and next to the left ventricle. Moreover the leptomeningeal spread progressed. The chemotherapy regimen was adapted to the HIT-REZ 2005 study and etoposide was now administered intraventricularly. However tumor control was not achieved. Shortly before his death, 10 months after the initial diagnosis, the boy developed pleural effusions, initially on one side and then bilaterally. Pleural effusions required pleurocentesis revealing predominantly malignant cells. From these pleural effusions, the cell line named MB3W1 (for medulloblastoma-Group 3-Würzburg 1) was derived.

### MB3W1 cells phenotypically and functionally display characteristics associated with CSC

**Fig. 2:**
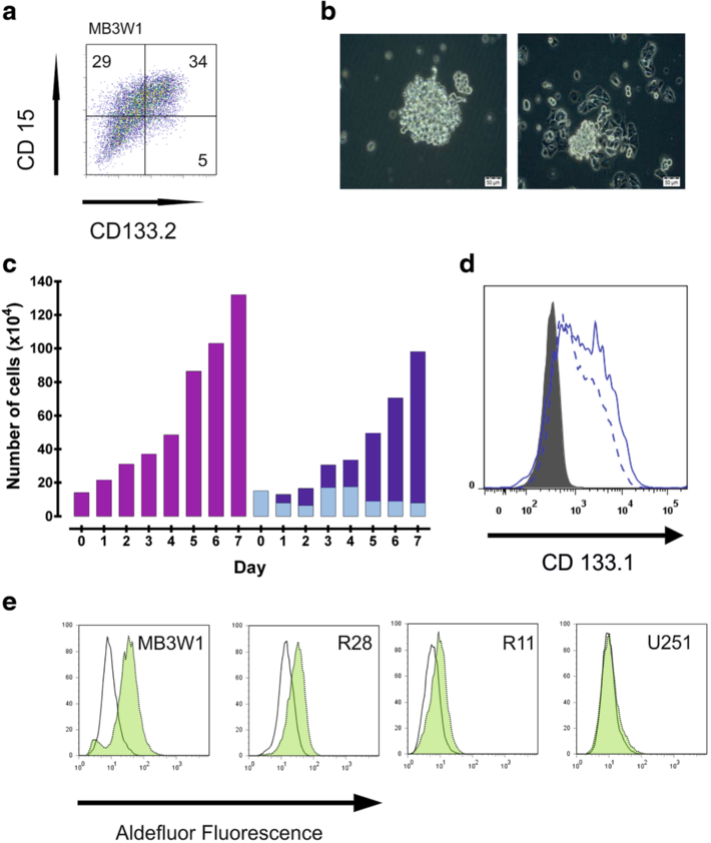
Characteristics of medulloblastoma cells recovered from the pleural effusions after progression of disease. **a**. Representative flow cytometry plots outlining the expression of CD133 and CD15 in MB3W1 cells. **b**. Light-microscopy of MB3W1 cells in culture in serum-free medium showing sphere forming growth (*left*) and partly adherent growth in serum-containing medium (*right*). **c**. Growth characteristics of MB3W1 cells grown in serum-free medium (left, *purple columns*), compared to growth in serum-containing medium (right, light blue columns = adherent fraction, dark blue columns = spheroid fraction). **d**. Expression of CD133 on cells grown as spheres in serum-free medium at in vitro passage 1 (*blue full line*) and passage 30 (*blue broken line*). Black line = isotype control. **e**. ALDH expression as measured by substrate conversion using the ALDEFLUOR assay. Different cell lines were incubated with ALDH-substrate either without (*green lines*) or with a specific enzyme inhibitor to block ALDH-activity (*black lines*). The degree of fluorescence correlates with ALDH-activity. R28 and R11 are glioblastoma cell lines recently characterized as cells with CSC activity. U251 is a glioblastoma cell line with no known CSC activity

After isolation of MB3W1 cells via Ficoll gradient and brief culture (4 days) in serum-containing medium, cells were analyzed by flow cytometry. We stained the cells for several markers associated with CSC [41]. Interestingly MB3W1 cells strongly expressed CD133 and CD15 (Fig. 2a), both of which are markers associated with CSC [37, 42–46]. Expression of these markers was high, when compared to other cell lines – from other tumor entities – with a documented CSC activity (glioblastoma: R28 and R11 [41]; breast cancer: MCF7 [47]; Additional file 1: Figure S1). Considering the phenotype, we asked whether cell growth could be maintained under culture conditions propagating neural stem cells. Culture in serum-free medium containing bFGF and EGF promotes the growth of undifferentiated stem cells that form neurospheres and show self-renewal and exponential long-term proliferation [42, 48, 49]. In fact, when MB3W1 cells were cultured under these conditions, they formed neurospheres (Fig. 2b) and proliferated rapidly (Fig. 2c). Long-term cultures were easily established. Cells could be passaged more than 30 times without showing changes in their growth characteristics. Flow cytometry analysis for CD133 after 30 passages showed no significant changes compared to the initial staining pattern (Fig. 2d). When cells were cultured in serum-containing medium, growth characteristics changed: cells became plastic adherent and proliferation decreased. However, neurosphere formation still continued. These neurosphere-building cells were the proliferating part while the adherent fraction basically did not proliferate (Fig. 2b and c).

We also analyzed ALDH activity in this cell line as we suspected CSC properties. Besides certain cell surface markers, ALDH activity is considered as a functional hallmark of CSC [50, 51]. MB3W1 cells displayed strong ALDH activity, which by far exceeded the activity of the previously described glioblastoma CSC lines R28 and R11 [41] (Fig. 2e). These findings indicate, that the majority of the primary cells from the pleural effusions show characteristics described for CSC.

### MB3W1 cells show aggressive orthotopic tumor formation in immunocompromised mice

**Fig. 3:**
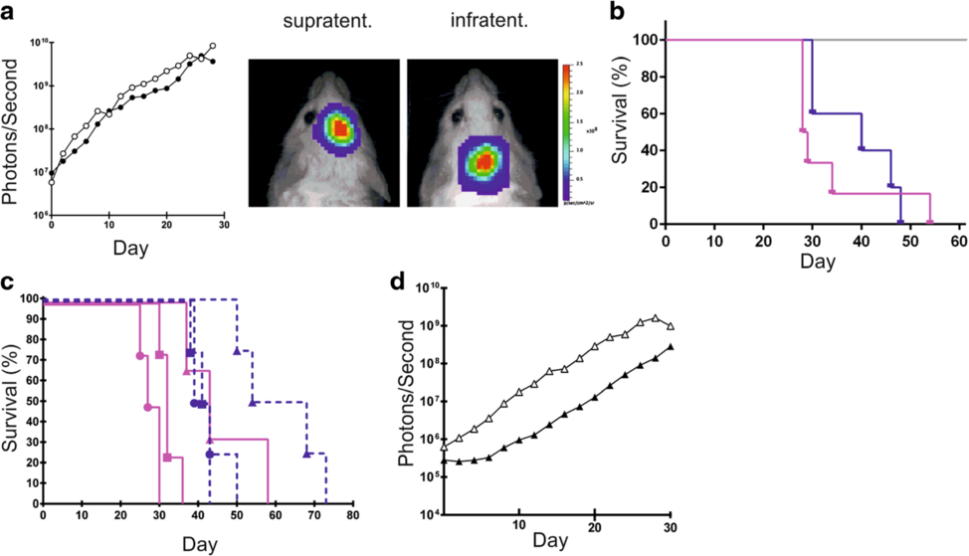
In vivo growth characteristics of MB3W1. **a**. 5 × 10^4^ luciferase-transduced MB3W1 cells were xenografted either infra- (*open circles*) or supratentorially (*closed circles*), and tumor growth was monitored by BLI. **b**. Survival curves after infra- (pink, *n* = 6) versus supratentorial inoculation (blue, *n* = 5; one animal lost during surgery (censored)); grey: untreated controls, surgery only (*n* = 2). **c**. Survival curves of mice inoculated with untransduced tumor cells: comparison of MB3W1 cells previously grown in different culture conditions: supratentorial inoculation of cells previously grown as spheres in serum-free medium (*pink curves*, *n* = 5) and compared to inoculation of cells previously grown in serum-containing medium – cells were only derived from the non-proliferative adherent fraction - (*blue curves*, *n* = 5). Circles: 5 × 10^5^ cells, squares: 5 × 10^4^ cells, triangles: 5 × 10^3^ cells. **d**. Growth characteristics of CD133^+^ (*closed triangles*, *n* = 5) and CD133^−^ (*open triangles*, *n* = 5) MB3W1 cells after supratentorial inoculation of 5000 tumor cells

CSC are highly tumorigenic when transplanted into immunocompromised mice. To directly examine this feature, MB3W1 cells were transduced with a lentiviral vector encoding FLuc and eGFP (data available on request). Transduction did not alter growth characteristics nor did it change the cells phenotype. When 5 × 10^4^ cells were injected either infra- or supratentorially into NOD/SCID mice, 100 % of tumors engrafted and grew immediately after inoculation (Fig. 3a and b). Survival was not significantly different, when implanting unmodified tumor cells. We next asked, whether growth differed, when tumor cells had been cultured in serum-containing medium, leading to a mixed population of adherent cells and small neurospheres in vitro. Tumor cells were injected supratentorially using titrated cell numbers ranging from as few as 5000 up to 5 × 10^5^ cells. Under either condition and with as few as 5000 cells per injection, tumor growth rates were 100 %. If tumor cells had been partially differentiated prior to transplantation using serum-containing medium (12 days), tumor growth slowed down, but still all animals developed tumors (Fig. 3c).

We next asked, whether in vivo growth characteristics differed when separating out CD133^+^ from CD133^−^ tumor cells. Separation was performed with magnetic beads, resulting in purities of 88.4 % for the CD133^+^ and 95.2 % for the CD133^−^ cell fraction. Both CD133^+^ as well as CD133^−^ cells remained highly proliferative with rapid tumor growth even at numbers as low as 5000 cells per injection, confirming that CD133 alone does not sufficiently define tumor initiating cells (Fig. 3d).

### Xenotransplanted MB3W1 cells exactly recapitulate the original tumor

**Fig. 4:**
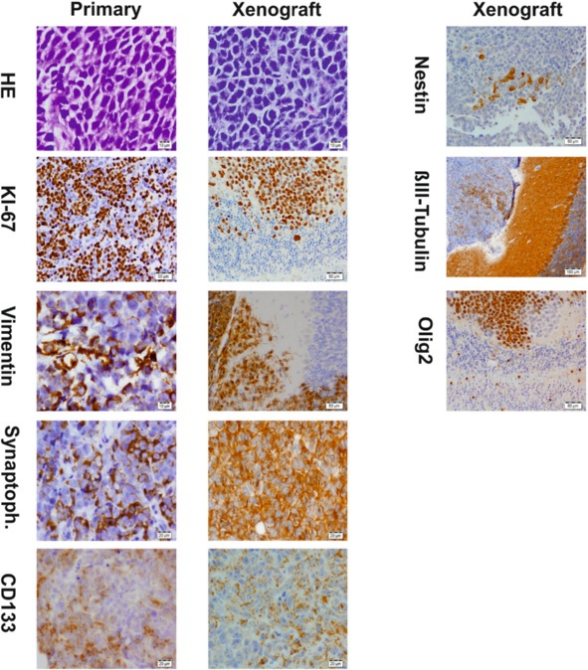
Immunohistological evaluation of the original tumor and xenografted infratentorial tumor. Selected markers are shown in comparison. For some markers, there was not sufficient material left from the primary tumor. All specimens were formaldehyde fixed and paraffin embedded prior to histopathological evaluation

One of the key criteria of CSC is that xenotransplants exactly recapitulate the histopathological characteristics of the original tumor [37]. We therefore compared xenotransplanted tumors with the histology of the patient’s primary tumor. Mouse xenografts exactly matched the morphological and biological characteristics of the original tumor. Xenotransplants showed anaplastic cell morphology consisting of tumor cells with marked nuclear polymorphism, typical nuclear angulation or moulding and frequent cell wrapping phenomena. Apoptosis (even whole areas of apoptotic cells) as well as necrosis were observed frequently. Abundant mitosis and a high Ki-67 staining index (50–60 %) reflected the high proliferation rate of MB3W1 cells (Fig. 4). When we examined tumors for their differentiation we found MB3W1 cells immunoreactive for markers known to be expressed by stem/progenitor cells (like CD133 and Nestin) [52, 53] and for markers associated with neuronal (like Synaptophysin and ßIII-Tubulin) [54, 55], oligodendroglial (like Olig2) [56, 57] or immature astrocytic differentiation (like Vimentin) [58] (Fig. 4). A mature astrocytic differentiation of tumor cells (indicated by GFAP) [59] could not be detected. Thus the experimental MB3W1 tumors match the original tumor in many histological and immunohistochemical features. Broad expression of lineage markers supports the potential of multilineage differentiation as typically observed in CSC.

**Fig. 5:**
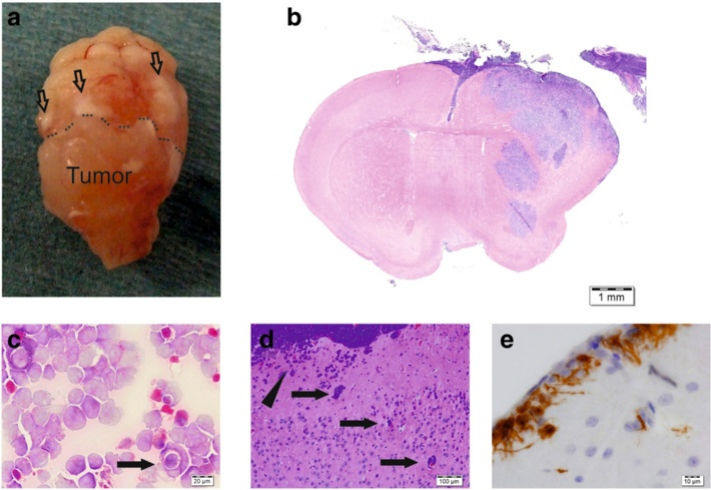
Xenografts show signs of metastasis. **a**. Macroscopically subarachnoidal tumor dissemination is seen frequently at tumor progression and **b**. tumor cells can then constantly be found in the CSF (HE stained gross section). **c**. Xenograft cytospin of subarachnoidal disseminated tumor cells at the humane endpoint reveal blue-cell tumor cells with for LCA histology typical cell wrapping (*arrow*). **d**. Histology (HE-staining) indicates aggressive, infiltrative behavior with xenografted tumor cells invading from the brain surface (*arrow head*) and the Virchow-Robin spaces (*arrows*). **e**. Vimentin-staining showing tumor cells with pseudopodia reaching from the brain surface into the brain tissue

Of note, MB3W1 cells also seem to reflect the invasive and spreading behavior of the patient’s tumor. At the humane endpoint, when tumors had grown to the maximal tolerable size, dissemination into the subarachnoid space could routinely be detected (Fig. 5a–c). Tumor cells present in the CSF displayed the same high proliferation as the cells that engrafted in the brain (proved by Ki-67 staining). Disseminated cells were also highly invasive. Cells invaded from the brain surface and from Virchow-Robin spaces into the brain (Fig. 5d and e) generating metastases even at sites far away from the initial inoculation point. Although technically there is a chance of artificial contamination of the CSF during the inoculation process, we believe that metastasis is rather due to the highly malignant characteristics of these tumor cells: we never detected such aggressive behavior when using other tumor cells. Furthermore leptomeningeal spread was an event occurring in a late stage of tumor progression, whereas mice sacrificed at earlier time points (1–2 weeks after transplantation) did not show any metastasis.

### Original pleural carcinosis showed a similar pattern of CD133^+^ as cultivated MB3W1 cells

**Fig. 6:**
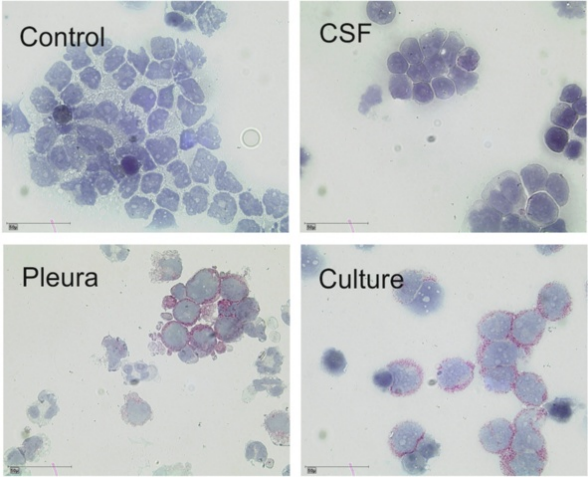
Evaluation of CD133 expression in MB3W1 cells directly after cell isolation and during cell culture. Cytospins from different cell preparations were evaluated for CD133. Preferred expression of CD133 on cells directly derived from the pleural effusions (*lower left*) and on cultivated MB3W1 cells at day 4 after isolation (*lower right*). In comparison cytospins from the CSF at diagnosis showed little (if any) staining for CD133 (*upper right*). (*Upper left*: staining control)

Extracranial metastasis of medulloblastoma, especially with intensified chemotherapy, is a relatively rare event [60]. In our case, cultivated tumor cells highly expressed CD133. Thus we asked whether this expression had occurred due to selection in vitro, or whether there had been a biologic enrichment of such undifferentiated cells during the course of disease progression. Enrichment during the 4 day culture prior to the first analysis by flow cytometry seems highly unlikely. More importantly immunohistochemistry of cytospins obtained from cells directly isolated from the pleural effusions showed a similar pattern of CD133 expression compared to the cultured tumor cells. In contrast, staining of the CSF cytospins, obtained at the time point of the initial diagnosis, revealed plenty of tumor cells, but CD133 expression was low (Fig. 6). This suggests that CD133^+^ cells had likely been the driving force for generating extracranial metastasis, emphasizing the highly aggressive and self-renewing characteristics of these cells.

### MB3W1 cells show properties associated with Group 3 medulloblastoma

**Fig. 7:**
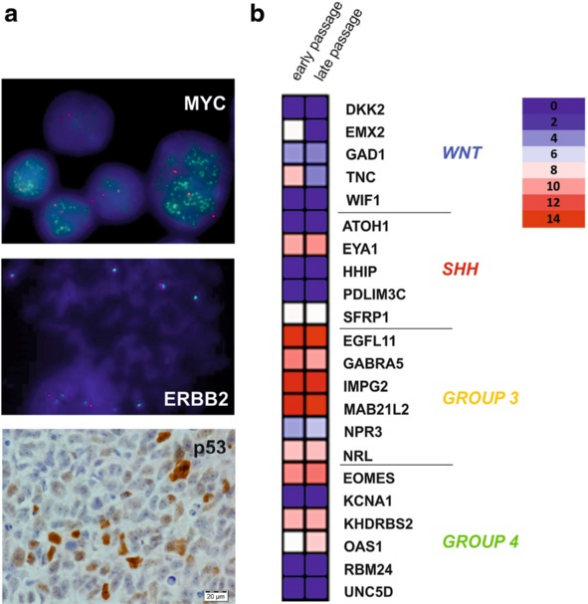
MB3W1 cells comprise a faithful Group 3 model. **a**. Upper panel: FISH analysis of MB3W1 cells revealed a clear amplification of the *MYC* oncogene (*green signal*) as a hallmark of Group 3 tumors (in red: *CEP8*). Middle panel: FISH on chromosome 17 shows (consistent with the patient’s karyogram) tetraploidy of the *ERBB2* gen (*red sig*nal) and an unbalanced gain of chromosome 17q (green signal of the chromosome enumeration probe 17p11.1-17q11.1). Lower panel: Immunohistochemical staining of xenotransplanted tumor specimen revealing accumulation of p53 protein. **b**. Heatmap, illustrating analysis of a set of 22 marker genes previously validated for MB sub-grouping. Strong clustering of genes regulated in Group 3 MB is observed. No significant differences in gene expression were detected in RNA derived from early passages (<5 passages) or late passages (>20 passages)

Among the recently identified molecular subgroups, Group 3 medulloblastoma have the most aggressive tumor biology. These tumors often exhibit features known to be associated with poor clinical outcome such as LCA histology, high dissemination tendency, gain of chromosome 17q and *MYC* amplification [5, 9, 18, 23, 24]. These are the same features observed in our patient’s tumor. Aberration of the *MYC* oncogene is one of the key molecular pathways in Group 3 medulloblastoma [25]: *MYC* can induce proliferation as well as apoptosis [61]. Because *MYC* induced apoptosis often depends on *TP53* function [62], alterations of *TP53* can compensate the apoptotic effect of *MYC* leading to enhanced proliferation of cancer cells [25]. Indeed in MB3W1 cells *MYC* was uniformly amplified and also immunohistochemistry showed p53 accumulation (Fig. 7a). Classic karyotyping (not shown) and FISH revealed a male, tetraploid chromosomal pattern with an unbalanced gain of chromosome 17q (Fig. 7a). All of these characteristics are often observed in Group 3 tumors [24, 63].

Furthermore using nanostring analysis of a set of 22 genes, which has been described recently to accurately define the molecular subgroups [40], we clearly confirmed that these tumor cells belong to the subgroup 3 (Fig. 7b). Comparative analysis of early and late passages of the cells showed little variation of gene expression, thus indicating a relatively stable gene expression pattern.

## Discussion

We here established a xenograft model for anaplastic medulloblastoma with a molecular Group 3 signature, which clinically has a very poor prognosis [18]. Therefore there is a clear need for additional animal models to study this tumor subgroup [64]. Only recently two groups established syngenic mouse models by genetically interfering with the *MYC* and *TP53* pathways, that mimic Group 3 characteristics. Pei et al. and Kawauchi et al. both introduced *Myc* into murine cerebellar cells by genetic engineering, which, in combination with p53 blockade (either by introducing dominant negative p53 into CD133^+^ cells of the cerebellar white matter or by using *Trp53* null granule neuron precursors) led to the formation of medulloblastomas resembling the Group 3 subtype [25, 26]. Our model is complementary to this work, as it recapitulates the orthotopic growth of highly aggressive human medulloblastoma without additional genetic engineering. The only modification of the tumor cells has been transduction with FLuc and eGFP for better monitoring. This modification does not change the biologic behavior of the cells, as in vitro growth (not shown) and survival of mice were identical. Milde et al. recently described a human Group 3 cell line, HD-MB03, focusing on the impact of HDAC-inhibitors as a potential treatment option [65]. In an evaluation of established long-term cultured cell lines, Shu et al. reported on good in vivo growth characteristics of D283-MED, a medulloblastoma cell line, that has some characteristics, albeit not complete congruency, of a Group 3 tumor cell line [66, 67]. Mastronuzzi et al. recently reported on a similar case of anaplastic medulloblastoma with metastasis to the scalp, also displaying some features of CSC in their in vitro evaluation [68]. Taken together, these unmodified human tumor models will advance the field of medulloblastoma research especially with respect to the dismal Group 3 tumors: a comprehensive analysis of the available Group 3 cell lines, HD-MB03 and MB3W1, plus potentially D283-Med, may lead to urgently needed new treatment strategies for this tumor type.

Further studies are necessary to determine whether this tumor model can also be used to study the mechanisms leading to metastasis: the data presented here suggest that metastasis into the CSF is characteristic for these cells once the tumor reaches a certain size. However there remains a slight chance of a potential contamination during the injection process. On the other hand, the dynamics of metastasis and our comparison to other transplanted tumor models strongly argue for spontaneous dissemination of the tumor cells. If so, this model will be extremely valuable to assess the effect of drugs targeting exactly this process of dissemination or to analyze the pathways leading to this malignant spreading.

What is the originating cell of MB3W1 cells? Much is known about the cells of origin and the driving pathways of WNT and SHH medulloblastomas, but Group 3 tumors are less well characterized [18–22]. Apparently cells of different brain compartments could lead to a Group 3 medulloblastoma as suggested by the two recently published murine models. Importantly, MB3W1 cells also display several characteristics of CSC, as described earlier for many tumor types [69]. The fact that xenotransplanted MB3W1 cells engrafted to 100 % with tumors exactly recapitulating the original tumor architecture, display functional characteristics such as high ALDH activity, neurosphere formation and exponential long-term proliferation all argue for stem-cell like properties [42, 48–51]. The expression of markers such as CD133 and CD15 also is suggestive for stem-cell like properties, although CD133 expression alone does not define this distinct population. This is in line with work from different groups, indicating that CD133^−^ tumor cells may also have CSC capacities [41, 44].

Extracranial metastasis in medulloblastoma is a relatively rare event. In the aforementioned case report, medulloblastoma metastasis in the scalp was observed and these cells also contained features of CSC [68]. The increase in CD133+ cells in in the pleural effusion in our patient, as well as the detection of CD133+ cells in the metastasis of this other report, may argue for a role of stem cell activity in the pathology of metastasis. It is unlikely, that the ventriculoperitoneal shunt facilitated the spread in our patient, as, despite pleural effusions, there was no documented peritoneal spread. As this is a singular case, we cannot determine whether CD133+ expression (and CSC-capacities) is the cause for progression and pleural spread or just coincidental. However the enrichment in the pleural effusions is indicative of biologically aggressive behavior of cells with this phenotype. Analysis in larger patient cohorts is necessary to potentially link this phenotype to clinical outcome.

Thus we conclude that the cells from which the MB3W1 cell line originated must have had the capacity to: 1) withstand chemo- and radiotherapy, 2) retain the molecular/histochemical characteristics as a highly aggressive, tumor initiating cell and 3) may have selectively crossed the blood-brain barrier, albeit in the context of a heavily pretreated patient, to disseminate to the pleura.

## Conclusions

The high percentage of cells with CSC characteristics within this cell line, which emerged after a natural selection process of extracranial metastasis, is remarkable and provides a unique tool for medulloblastoma research. We believe that this tumor model will be extremely valuable to study the aggressive biological behavior of Group 3 medulloblastoma, - with a focused approach to CSC, as well as to explore possible therapeutic interventions. MB3W1 cells will be made available upon request (to Wölfl M or Schlegel PG).

## Consent

Written informed consent was obtained from the parents of the patient for publication of this case report and any accompanying images. A copy of the written consent is available for review by the Editor of this journal.

## Additional file

Additional file 1: Figure S1. Phenotypical comparison of MB3W1 to other tumor cell lines.ᅟ (JPEG 2532 kb)

## Abbreviations

ALDH: Aldehyde dehydrogenase
AT/RT: Atypical teratoid/rhabdoid tumor
bFGF: Basic fibroblast growth factor
BLI: Bioluminescence imaging
CSC: Cancer stem cells
CSF: Cerebrospinal fluid
DNA: Deoxyribonucleic acid
EGF: Epidermal growth factor
eGFP: Enhanced green fluorescent protein
EWS: Ewing sarcoma
FLuc: Firefly luciferase
HE: Hematoxylin eosin
LCA: Large cell anaplastic
MB3W1: Medulloblastoma Group 3, Würzburg 1 cell line
MRI: Magnetic resonance imaging
RNA: Ribonucleic acid
RT: Room temperature
SHH: Sonic hedge-hog
WNT: Wingless/Int-1
