# Supplementary figures and images for: MB3W1 is an orthotopic xenograft model for anaplastic medulloblastoma displaying cancer stem cell- and Group 3-properties
Source: BMC Cancer. 2016 Feb 17;16:115. doi: 10.1186/s12885-016-2170-z (PMC4756501; doi:10.1186/s12885-016-2170-z)

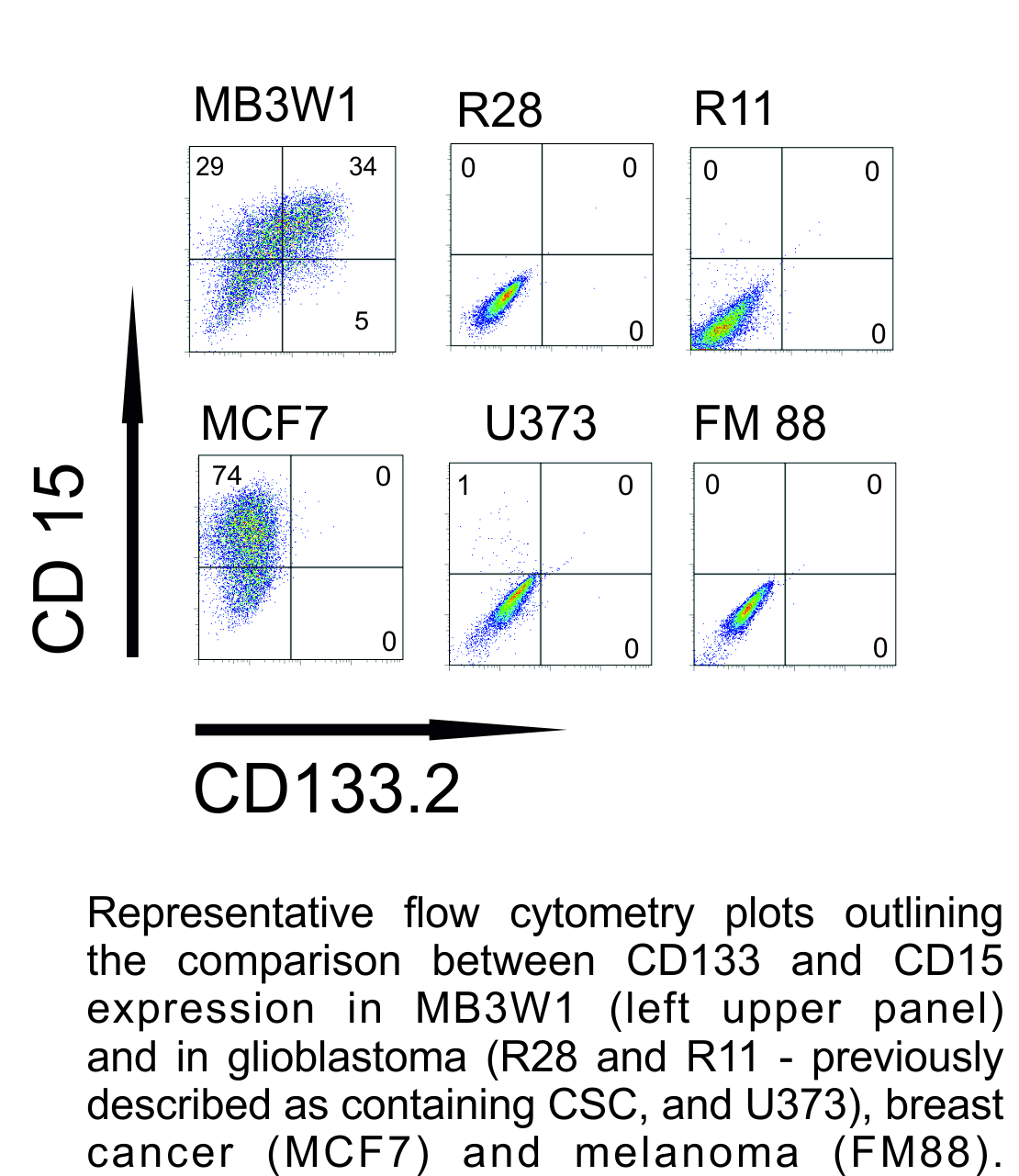

Supplement: Additional file 1: Figure S1. — Phenotypical comparison of MB3W1 to other tumor cell lines.ᅟ (JPEG 2532 kb) [file 12885_2016_2170_MOESM1_ESM.jpeg]
